# A Report of Four Surgical Cases*A paper read, with presentation of specimens, before the Dallas Medical and Surgical Association, December 29, 1900.

**Published:** 1901-02

**Authors:** Henry K. Leake


### TEXAS MEDICAL JOURNAL.

ESTABLISHED JULY, 1885.

PUBLISHED MONTHLY.—SUBSCRIPTION $1.00 A YEAR.

VOL. XVI. AUSTIN, FEBRUARY, 1901. No. 8.

### Original Contributions.

### A Report of Four Surgical Cases.*

BY HENRY K. LEAKE, M. D.

At the last meeting of the District Clinical Society I presented the history of five recent interesting cases with accompanying specimens.

From a list of subsequent operations I have selected the records of four cases equally interesting, which I desire to present before this society. The specimens are also offered for inspection and study. I believe that some useful lessons may be drawn from a full discussion of these cases.

DISLOCATED AND ADHERENT RIGHT KIDNEY; HYDRONEPHROSIS; NEPHRECTOMY.

This patient was referred to me by Dr. King, of Collin county, and I visited her at her home. Mrs. D., age 26; married, five children, ages 3, 5, 7, 9 and 10 respectively; no miscarriages; brunette, medium height, well developed, weighing about 130 lbs., menses regular, painless, but rather profuse. She was in bed convalescing from an assumed attack of mild malarial fever. She was very despondent and taciturn. I was informed that some months previous she had been subject to slight mental aberrations, approaching a true type of melancholia.

*A paper read, with presentation of specimens, before the Dallas Medical and Surgical Association, December 29, 1900.

During one of her attacks she had left home to walk several miles to her father's for the purpose of abandoning her husband, for whom she had conceived a great aversion without cause. She said that she had a lump in her right side which was the cause of all her trouble; the lump was first observed eight years before. On examiation this lump was discovered. It was of reniform shape and presented in an oblique direction below the right hypochondrium. The center of its lower border was on a level with the umbilicus. It had an obscurely elastic and almost solid feel. By strong pressure it could be moved toward the loin, but also upward under the liver. On removal of the pressure it resumed its original position with a quick rebound. Pressure gave much pain and caused nausea. There was no corresponding tumor on the left side. I obtained a somewhat indefinite history of intermittent discharges of urine.

The patient stated that on walking she was, on account of pain, compelled to support the lump with her hands. She had been extremely nervous; gastric symptoms were slight, headaches were not prominent, nor was there much heart disturbance. She was anemic, bowels were fairly regular. The uterus was retroverted to the second degree, but not markedly enlarged. Appendages were normal. There was slight endometritis. Other abdominal organs were not displaced.

Diagnosis: Dislocated, enlarged and adherent kidney. On entering my private hospital, October 2, 1900, by means of the Harris instrument, it was shown that there was discharge from both ureters into the bladder; examination by Dr. Shelmire discovered nothing abnormal in the urine.

October 9th, the uterus was thoroughly curetted, and after this until October 15th the urine was repeatedly and carefully examined. On the latter date operation was done. On exposing the kidney through a lumbar incision it appeared as a lobulated tense-walled cyst. The organ was turned on its axis, the upper pole looking towards the spleen, the right towards the lower lumbar region. It was tapped and withdrawn through the incision. On examination Dr. Graham concurred in the opinion that its removal was necessary. The ureter was fixed in the lower angle of the incision, the renal artery and vein were tied off with catgut and the organ removed from its peritoneal adhesion. This was cautiously done, but left a long rent in the parietal layer of the peritoneum, exposing the right lobe of the liver. This rent was closed by a continuous suture of fine catgut, the remaining cavity lightly packed with gauze, followed after a few days by drainage tube. Healing was complete in three weeks, when patient left for her home. She writes that she is wonderfully improved in all respects, and is rapidly returning to the condition previous to marriage.

Standard surgical authority has formulated the rule that a kidney may be removed under the following conditions: (1.) Benign or malignant tumor; (2.) Pyelitis; (3.) Tuberculosis. Are there no exceptions to this rule? Clearly it was violated in this case. Ascertaining that the opposite kidney was sound and functionating, that draining a cystic kidney might subject a patient, weakened as this one was, to a prolonged suppuration and that nephropexy, with or without drainage, will often fail, removal of the kidney was considered justifiable. On the other hand some clinicians assert that one kidney being removed the additional strain thrown upon the other renders it very liable to future disease. I am not informed as to the character of the disease meant, as I have had no experience in observing the future of these cases. Will you fix the cut ureter into the lower angle of the incision to provide for possible reflux of urine from the bladder?

For anatomical reasons, supported by actual experiments on the cadaver, it is denied that urine will ever back up the ureter, even in the worst cases of urethral stricture. In a recent case of nephrectomy by the anterior abdominal incision, the ureter was securely tied with fine silk and dropped. The patient has been watched since; no ureteral distension has been ascertained, nor has there been any local septic infection from either the ligature or the stump. If the latter be feared, the stump must be left short and touched with the Paquelin cautery. In the case above reported the ureter was fixed, but I consider this to have been unnecessary.

### UTERINE FIBROMA; FIBROMA OF THE LEFT FALLOPIAN TUBE; UTERINE FIBROMA; CYSTOCELE; ABDOMINAL HYSTERECTOMY; ANTERIOR COLPORRHAPHY.

Mrs. K., of Dallas, age 46, weight 156 lbs., tall, fat and muscular, married, 2 children, ages 12 and 20 years respectively. Had never miscarried. Her occupation kept her almost constantly on her feet.

Since the birth of her first child she has suffered intensely from bladder symptoms, for which she has had many different kinds of treatment, both local and general. She said that her bladder was always down between her thighs when she stood up, and she often palpated the neck of her womb at the vaginal entrance. Her menstruation for the past year had been regular, but very profuse, amounting to actual hemorrhage, the consequences of which were apparent in her somewhat bluish lips and anemic countenance. Her last period, which had just passed, had lasted over ten days, with a larger flow than usual. Several months ago she had been informed by a physician that besides the cystocele and uterine prolapse, she had an intra-uterine fibroid.

She entered my private hospital Nov. 1, 1900. There was a large cystocele, the size of a hen's egg, the cervix presented at the introitus vagina, the uterus measured 4½ inches, was movable and could be elevated by the finger. The remainder of the pelvis seemed free from disease. The opinion of the previous physician was confirmed and operation advised.

On Nov. 4, 1900, abdominal hysterectomy was performed in the Trendelenburg position, the uterus being removed from the internal os.

The cervix was trimmed down wedge-shaped fashion on a level with the latter. The cervical flaps were apposed by catgut sutures, the peritoneum drawn and sutured over the stump and the open spaces in the broad lingament on both sides were united by a semicircular line of fine catgut; thus closing all raw surfaces by peritoneum left within the pelvic cavity. The fat apron was then brought down and carefully tucked over the entire roof of the pelvis, the abdominal incision being closed by tier suturing with catgut and silkworm gut. After this the patient was lowered to the lithotomy position and anterior colporrhaphy performed. Ether was the anesthetic administered.

There was some shock, but reaction promptly followed. Immediately thereafter the most violent retching and vomiting which I have ever witnessed ensued. For four days and nights this was incessant, the patient rolled and tossed in every conceivable manner, no amount of persuasion, or even force, being sufficient to restrain her movements. Nothing whatever could be retained; she vomited immense quantities of ropy mucous. With the greatest difficulty could she be induced to try stomach remedies; she positively refused to allow opiates in any form, my promise being rigidly exacted that I would withhold them, no matter what the consequences. The urine examined before the operation was normal, it now contained 25% albumen and was scant; she passed three ounces only the first 24 hours after the operation. Under protest, we were allowed to persist with several doses of calomel, followed by large enemas of water to which some turpentine was added, notwithstanding the condition of the kidneys. The bowels moved freely with no effect upon the retching and vomiting. We finally compelled her to submit to stomach lavage with several pints of deci-normal salt solution; this gave the first relief, but she could not be induced to try it again.

Strychnia hypodermics were systematically given, also enemas of normal salt solution and panopepton. This condition continued for two weeks, when she began gradually to keep down small quantities of panopepton, together with Buffalo lithia water. The urine was now free from albumen. After a few days she partook of other light food, but soon a lienteric diarrhea, which became colliquative, ensued. All food now passed the bowels unchanged, and the patient was constantly weakening. The pulse became extremely fast and feeble, reaching 130 or more. The temperature seldom rose above 100°, on one occasion only reaching 102°. The maximum temperature was caused by the formation of a stitch abscess, which occurred through infection of the lowest silkworm stitch, due to the constant agitation of the patient, exposing at times this part of the incision.

The abscess healed within a few days and the temperature lowered to normal. Finally, as a last resort, the patient submitted to suppositories of opium and large doses of bismuth by the mouth, which, being retained, controlled the bowels. Stomach and intestinal indigestion, however, persisted; this was much relieved by the administration of Sharp & Dohme's preparation of pepsin and pancreatine with hydrochloric acid. The patient gradually emerged from this serious condition and at the end of five weeks had regular bowels, a voracious appetite and improved spirits. All her wounds had entirely healed. At the end of the fifth week she was suddenly seized with a distinctly malarial chill followed by fever. This promptly yielded to quinine, and shortly thereafter she left the hospital for home with a slight remnant of bladder irritation, caused doubtless by several days retention in the bladder of a Jacob's catheter, which had been introduced after the operation. For this irritation before leaving the hospital, the bladder was irrigated with permanganate of potash solution, 5 grains to the pint, and later by the employment of boric acid solution, which gave great relief. After her return home, irrigation of the bladder was continued, a solution of oil of cloves in sterile water in the proportion of one part to 12,000, or one drop to the pint, being employed. This answered well.

I ascribe my troubles in this case to the protracted administration of ether, the patient having been under this anesthetic just two hours and twenty minutes—the longest time consumed in any operation which I have done; the hurtful and unnecessary expenditure of time being due to overlooking a severe anastomotic artery in the left broad ligament close to where the uterine was ligated. More than 30 minutes were required in the search for and the securing of this actively bleeding vessel, which might as well have been attended to before the removal of the clamp. Lapthorn Smith had an accident with a severed right uterine artery, his patient within a few minutes dying upon the table. But I saw Lawson Tait under similar trying circumstances; the lightning-like skill of the great thaumaturgist rescued the patient.

The specimen affords a rare example of broad ligament fibroid, probably originating in the muscular tissue of the left Fallopian tube.

Fibroma of the broad ligament was originally held to take its origin invariably from the uterus by a pedicle, which finally breaking away by attenuation or otherwise, the tumor then grows independently. Pozzi, however, asserts that "Sanger, Biefinger and Freund have demonstrated the autogenoue origin in cases upon which they operated"; that is, originating from the connective and muscular tissue of the leaflets of the broad ligaments. I have enucleated several such growths. The tubes are but extensions of uterine tissue, and thus afford conspicuous histological basis for these growths in rare cases. If the fibroma be in the ovary, which is also a rare location, it may derive its origin from the muscular tissue of the tubules of the par-ovarium which extend into the ovarian tissue proper.

We are indebted to Koch for a new remedy in the treatment of various forms of cystitis. He had found that oil of cloves "crippled" the bacilli in tubercuolus cases. German surgeons utilize this idea in the treatment of other kinds of cystitis, particularly that following the use of catheters. In this class of cases Dr. Goldspohn, of Chicago, highly recommends it.

I can not close this report without urging the great importance of stomach lavage in serious cases of vomiting after laparatomy. Humiston says "The results were so striking and immediate in two desperate cases, after failure to control vomiting by every known method, that hereafter I shall advise washing of the stomach thoroughly an hour before operation in cases that have been troubled with gastric irritability for a length of time." But stomach lavage must ordinarily be persisted in. It was utterly impossible to induce my patient to allow this.

CARCINOMA OF THE UTERUS; VAGINAL HYSTERECTOMY.

Through the kindness of my friend, Dr. McKnight, of Mansfield, this patient applied at my hospital for treatment on Dec. 11, 1900. She had previously consulted two eminent Texas surgeons, who gave her slender hopes from operation. Mrs. R., age 50, weight 140 lbs., stout and muscular, no cachexia, no involvement of glands anywhere. Married 34 years, had four children, the youngest being 24 years old, no miscarriages. She was never in labor more than two or three hours, and was invariably out of bed on the second or third day. Her menses continued with normal regularity and amount up to several months ago, when the flow became continuous, though not profuse nor bad smelling. Examination showed the introitus and vaginal walls free from disease, the cervix to the depth of half an inch or more was excavated by a ragged ulcer, which bled freely on the slightest touch. The ulceration had left little of the cervical lips remaining, so little that there was deceptive appearance of continuous vaginal wall being involved. The uterine body was enlarged, but movable; the cervical portion, however, was fixed, but no extra cervical induration in the broad ligaments could be detected to account for this. No other pelvic disease was discovered. Vaginal hysterectomy was performed by the clamp method on the 6th, great care being taken to keep the incision round the cervix well outside this point. Recovery uneventful.

I should have avoided operation in this case had the vaginal wall, or extra uterine structures been involved, as was probably the opinion of the surgeons above referred to. Even in that case the uterus might have been removed from above by Werder's method, but I have never been tempted to try his operation, and I do not know that I ever shall. The fixity of the cervix without special induration of the parts, the fundus being movable, does not always indicate extra-cervical extension of the disease. At this period of life shortening and rigidity of the ligaments, binding the cervix, will produce this effect. When suspicious I have dissected up laterally into the lower portion of the broad ligaments, and on finding these diseased, have abandoned total extirpation, amputated the remaining portion of the cervix and deeply cauterized the stump with the Paquelin cautery. Dr. Bourland of this society once assisted me in doing such an operation. Cancer, according to Lawson Tait and others, invariably has its origin in epithelial structures; cancer of the cervix-uteri will first attack either the squamuos epithelium of the vaginal portion of the cervix, the epithelium lining of the cervical glands, or the epithelium covering of the cervical canal between the openings of these glands. Now in the last two varieties the disease almost invariably extends up the uterine canal to attack the cavity of the uterus before extending to the broad ligaments. The specimen in my case shows the carcinomatous disease belong to either one of these two varieties. My judgment was that the uterine cavity was not invaded, hence I felt safe in believing that extra-uterine tissue, including the ovaries, was not involved, and proceeded with the operation. In cutting away the uterus also, the scissors seemed to pass through normal tissue. Moreover, no pelvic glands could be felt, although they may have escaped a close examination. Of course, no one can say what the future prospects of the patient are, and she has been encouraged by no unwarranted promises. You will observe that the ovaries were not removed. In all my previous vaginal hysteretomies for cancer, I have done this and it is a good surgical rule to follow. In this case, however, the uterine body being somewhat large, the vagina narrow, the ovaries not felt to be enlarged on previous examinations and not appearing in the wake of the incision, and, further, to save time during the operation these organs were left in situ.

PAR-OVARIAN CYST; DERMOID; LIPOMA OF THE ABDOMINAL WALL SITUATED IN THE RIGHT ILIAC REGION.

This patient was sent to me by Dr. Ferris, of Henrietta, for whom, several years ago, I successfully operated on a niece of my lamented friend, Marion Sims, of New York. Mrs. B.—age 25, married 5 years, medium height, weight 160 pounds, with an enormous development of fatty tissue, particularly in the abdominal wall. She is the mother of one child 4 years old, no miscarriages; always healthy except that she was subject to catarrh of nose and throat; gradually growing stouter; menses normal. One year ago she observed a slight swelling in the right iliac region; this was supposed to be an ovarian cyst. I examined her on October 16, 1900. She came to my private hospital November 7. There was general protrusion of the abdominal walls due to the abnormal presence of adipose tissue. In the right iliac region this protrusion was slightly more in evidence. Under the strong palpation, through much overlaying fat, a mass was discovered of semi-elastic feel; it appeared obscurely oblong, apparently parallel to the right broad ligament. It gave slight movement under strong manipulation. It did not move on respiration; it did not in marked outline distinctly protrude the abdominal wall. On bi-manual palpation its inner border seemed to lie close to the uterus, but the sound with some difficulty moved the uterus to the left, thus demonstrating an hiatus between the two. The uterus was of normal depth and in normal position; no other pelvic abnormality was discovered. The tumor was considered to be either a broad ligament fibroma, or tense-walled intra-ligamentous cyst probably of ovarian origin. Operation was done on November 11th. The abdominal incision passed through four or more inches of fat, Dr. Graham remarking that it was the deepest abdominal incision he had ever seen. The omentum was loaded with masses of fat. Exploration by the hand discovered the mass projecting well into the right quadrant of the pelvis. The uterus was normal.

Examination of the right broad ligament recognized a par-ovarian cyst, the size of a mandarin orange; this was freely broken up by the hand, its pale fluid escaping into the abdominal cavity. Next was encountered a small dermoid the size of a walnut, growing by a lender style to the right ovary; this was pinched off and removed; it contained a mass of blonde hair and sebaceous matter. Withdrawing the hand, the sheath of the right rectus muscle was laid open to its outer border, when the tumor wall presented. It lay underneath a mass of fat, its under surface in contact with the fascia and peritoneum which had been pushed before it into the pelvic cavity, a very thin layer of the abdominal muscles remaining on its under surface to indicate their presence. The tumor was firmly adherent to its bed and had to be enucliated with great care to avoid buttonholeing at one or many points its peritoneal investment. The abdominal cavity was freely irrigated with salt solution; the abdominal incision was closed by tier suturing and a large hole cut into the tumor cavity just above the right ilium. The cavity was lightly packed with iodiform gauze, the ends of which were brought out through the counter opening.

Everything went well until the third day, when acute bronchitis throughout both lungs was diagnosticated. Embarrassment to breathing and cough was distressing. Meanwhile, the urine was passing in normal character and quantity. At noon of the fourth day there was sudden cessation of the excretion of urine. A Jacob's catheter was introduced into the bladder to ascertain if even a drop passed so that it could be examined. On the morning of the fifth day it was known that she had passed only two drams of urine during the preceding eighteen hours; it contained 50% of albumen. During the night the temperature of the room had been raised to 90°, a half dozen large dry cups were placed over the region of the kidneys, these were replaced by thick, hot flax seed poultices. The bowels had been moved the day before by several turpentine enemas, notwithstanding this, fifteen grains of calomel were administered, which acted freely. Under heavy blankets perspiration was copious. Towards the middle of the fifth day the urine began to increase in amount, also the percentage of albumen.

Acute colitis also, now supervened. About the tenth day the bronchitis and colitis had nearly disappeared. Three weeks thereafter, although the patient was excreting sixteen ounces or more of urine daily, the percentage of albumen had risen to nearly 90%. She was drinking freely of milk and Buffalo Lithia water, and the action of the skin was maintained.

The bowels were daily washed out with hot deci-normal salt solution. She did not show anasarca at any time, vision was perfect, there was no headache, stomach retentive, but she was apathetic and despondent. The heart action became rapid and weak. The abdominal incision had quickly healed. A second counter-opening was made just above the pubis and a large rubber tube introduced through both counter-openings to provide freer drainage from the mural tumor cavity. This condition of affairs continued up to the beginning of the fifth week, when the amount of urine reached thirty ounces and the percentage of albumen had lowered to about 5%. Etimated by Haeser's method, she was now excreting 699 grains of solid matter in the urine, but according to Etheridge's table of body weights the urine should have contained 1198 grains of solid matter. (This method should have been applied before, but was neglected. Certain it is that she had previous to this reckoning excreted solid matter through the kidneys in alarmingly small amounts.) Nevertheless, the patient now showed signs of improvement. On December 20th the urine was again examined; during the preceding twenty-four hours she passed 36 ounces of urine, specific gravity 1012; albumen about 3%; but curiously enough, Haines-Haeser's calculation showed only 419.4 grains of solid matter. Pulse was still rapid but improving in volume, temperature normal, as it had been throughout her sickness. On December 22d, she had voided during the previous twenty-four hours 46 ounces of pale urine,, specific gravity 1010, albumen 3% solid matter excreted amounting to 506 grains. December 25th, examination of the urine showed specific gravity 1008, solid matter excreted 387.2 grains in 44 ounces of urine passed during the previous twenty-four hours. The albumen had diminished to about 1%. The patient was then sitting up several hours during the day; she was sleeping and eating well, and her mind was not so cloudy.

Although the examination of the urine demonstrates the nephritis not yet relieved, with a strong probability of its drifting into a chronic condition, the patient will leave the hospital for home on December 29th, where treatment will be continued. There still remains slight drainage from the counter-openings.

Whence came this trinity of furies—acute bronchitis, acute desquamative nephritis and acute colitis—to mar the clinical picture of a case otherwise simple? Did they own a common cause, such as the malign effects of both anesthetics employed? for during the operation Dr. Whitis changed from ether to chloroform, remarking that she was taking the former badly. If only one of the anesthetics, which was the dire offender? Was her condition due to chilling of the body during the operation or afterwards? Concerning this last I may say that my assistants have always complained of the oppressive warmth of my operating room, and I can vouch for the care taken of my patients as to exposure upon the operating table and subsequently. Since the published emphasis of Turck enjoining the closest attention to details for the prevention of shock during operation, I have taken extraordinary precautions. Did the complications above mentioned originate from several causes? If the acute bronchitis and nephritis were caused by cold, or ether, or both combined, what originated the acute colitis? Was this caused by several turpentine injections employed to move the bowels? These injections amounted to three, each containing one ounce of turpentine to the quart of fluid. Furthermore, in considering these questions it should be remembered that this patient was the subject, so to speak, of a catarrhal dyscrasia.

I regret that in this case the urine was not examined for casts, but the presence or absence of casts and albumen or variations in the amount of urine passed, or the specific gravity, will only assist to solve the problem of nephritis, for one or all of these factors may be absent or misleading and yet nephritis may exist. Either singly or combined, as positive signs of nephritis they have fallen from their high estate in our confidence. Persistent renal inadequacy, as shown by the amount of solid material excreted in the urine, is now the crucial test, and if accuracy is desired, must be ascertained. Professor Davis, of Philadelphia, has emphasized this point with astonishing clearness, and in a letter to me gives many details in the diagnosis of renal inadequacy, all grouped about this central idea.

This patient was given hypodermic strychnia with some, but less caution that would have been the case under other circumstances.

Strychnia is in common abuse by the profession. In all septic conditions, whether or not following operations, as well as in typhoid and other febrile states where there is invariably a more or less granular, fatty or cloudy-swelling change in the heart muscle, the too liberal and protracted use of strychnia may be fraught with great harm to the patient.

Its misnamed tonic effects are secured by acting upon muscular irritability, thus "putting the whip to the already overworked horse." Latent or reserve force may thus be utilized, but this sooner or later, under the persistent use of strychnia, may be rapidly exhausted and the existing depression actually increased. As Professor Pearson, of Cork, has recently said with respect to the use of medicines in disease, it is better to rely solely upon the *vis medicatrix nature* rather than unwisely to interfere with those wonderful resources of nature which are so often sufficient to relieve the patient; thus reasserting the maxim left us by the father of medicine himself—Hypocartes—that "A physician if he cannot cure his patient will do him no harm." Strychnia adds nothing material to the economy; it has no counterpart in iron or arsenic, which have real hemolytic powers, themselves limited to their constructive and remedial benefits. It may be useful, therefore, in impending or acute cardiac failure where the heart muscle is little or not at all degenerate, but even here its prolonged use should be avoided, otherwise it can do no harm and is not the "balm in Gilead" it was once considered to be. In a correspondence which I have recently had with Professor Hobart Hare, of Philadelphia, on this topic, he sustains this reasoning, which I have long entertained, concerning the employment of strychnia in certain diseased conditions caused or attended by cardiac debility. Professor Hare also asserts that its injudicious use may induce fever of an irritative type, which much imperils the safety of the patient.

I have classified the solid tumor removed as fatty, although microscopically it hardly presents this appearance. It will be submitted to a histologist and a report of its character made to this society on a future occasion. The dermoid cyst was unfortunately misplaced, and cannot be shown.

I owe you an apology for the prolixity of this report, judged by ordinary professional custom.

An English writer has truly remarked that now-a-days medical men phrase their papers much as one would in placing his thoughts upon a telegraph form, thereby leaving many interesting points out of view. If you do not agree with his opinion, I, at any rate, have profited by his suggestion; but have doubtless wearied you in thus performing a duty to which I have been assigned.

